# Early Eculizumab Withdrawal in Patients With Atypical Hemolytic Uremic Syndrome in Native Kidneys Is Safe and Cost-Effective: Results of the CUREiHUS Study

**DOI:** 10.1016/j.ekir.2022.10.013

**Published:** 2022-10-18

**Authors:** Romy N. Bouwmeester, Caroline Duineveld, Kioa L. Wijnsma, Frederike J. Bemelman, Joost W. van der Heijden, Joanna A.E. van Wijk, Antonia H.M. Bouts, Jacqueline van de Wetering, Eiske Dorresteijn, Stefan P. Berger, Valentina Gracchi, Arjan D. van Zuilen, Mandy G. Keijzer-Veen, Aiko P.J. de Vries, Roos W.G. van Rooij, Flore A.P.T. Engels, Wim Altena, Renée de Wildt, Evy van Kempen, Eddy M. Adang, Mendy ter Avest, Rob ter Heine, Elena B. Volokhina, Lambertus P.W.J. van den Heuvel, Jack F.M. Wetzels, Nicole C.A.J. van de Kar

**Affiliations:** 1 Department of Pediatric Nephrology, Radboud University Medical Center, Amalia Children’s Hospital, Radboud Institute for Molecular Life Sciences, Nijmegen, the Netherlands; 2 Department of Nephrology, Radboud University Medical Center, Radboud Institute for Health Sciences, Nijmegen, the Netherlands; 3 Department of Nephrology, Amsterdam University Medical Center, Amsterdam, the Netherlands; 4 Department of Pediatric Nephrology, Amsterdam University Medical Center, Emma Children’s Hospital, Amsterdam, the Netherlands; 5 Department of Nephrology, Erasmus Medical Center, Rotterdam, the Netherlands; 6 Department of Pediatric Nephrology, Erasmus Medical Center, Sophia Children’s Hospital, Rotterdam, the Netherlands; 7 Department of Nephrology, University Medical Center Groningen, University of Groningen, Groningen, the Netherlands; 8 Department of Pediatric Nephrology, University Medical Center Groningen, University of Groningen, Beatrix Children’s Hospital, Groningen, the Netherlands; 9 Department of Nephrology and Hypertension, University Medical Center Utrecht, Utrecht, the Netherlands; 10 Department of Pediatric Nephrology, University Medical Center Utrecht, Wilhelmina Children’s Hospital, Utrecht, the Netherlands; 11 Department of Nephrology, Leiden University Medical Center, Leiden, the Netherlands; 12 Department of Pediatric Nephrology, Leiden University Medical Center, Willem-Alexander Children’s Hospital, Leiden, the Netherlands; 13 Department of Pediatric Nephrology, Maastricht University Medical Center, Maastricht, the Netherlands; 14 Dutch Kidney Patient Association, Bussum, the Netherlands; 15 Department for Health Evidence, Radboud University Medical Center, Radboud Institute for Health Sciences, Nijmegen, the Netherlands; 16 Department of Pharmacy, Radboud University Medical Center, Radboud Institute for Health Sciences, Nijmegen, the Netherlands

**Keywords:** atypical hemolytic uremic syndrome, complement, complement inhibition, cost-effectiveness, eculizumab, thrombotic microangiopathy

## Abstract

**Introduction:**

The introduction of eculizumab has improved the outcome in patients with atypical hemolytic uremic syndrome (aHUS). The optimal treatment strategy is debated. Here, we report the results of the CUREiHUS study, a 4-year prospective, observational study monitoring unbiased eculizumab discontinuation in Dutch patients with aHUS after 3 months of therapy.

**Methods:**

All pediatric and adult patients with aHUS in native kidneys and a first-time eculizumab treatment were evaluated. In addition, an extensive cost-consequence analysis was conducted.

**Results:**

A total of 21 patients were included in the study from January 2016 to October 2020. In 17 patients (81%), a complement genetic variant or antibodies against factor H were identified. All patients showed full recovery of hematological thrombotic microangiopathy (TMA) parameters after the start of eculizumab. A renal response was noted in 18 patients. After a median treatment duration of 13.6 weeks (range 2.1–43.9), eculizumab was withdrawn in all patients. During follow-up (80.7 weeks [0.0–236.9]), relapses occurred in 4 patients. Median time to first relapse was 19.5 (14.3–53.6) weeks. Eculizumab was reinitiated within 24 hours in all relapsing patients. At last follow-up, there were no chronic sequelae, i.e., no clinically relevant increase in serum creatinine (sCr), proteinuria, and/or hypertension in relapsing patients. The low sample size and event rate did not allow to determine predictors of relapse. However, relapses only occurred in patients with a likely pathogenic variant. The cost-effectiveness analysis revealed that the total medical expenses of our population were only 30% of the fictive expenses that would have been made when patients received eculizumab every fortnight.

**Conclusion:**

It is safe and cost-effective to discontinue eculizumab after 3 months of therapy in patients with aHUS in native kidneys. Larger data registries are needed to determine factors associated with suboptimal kidney function recovery during eculizumab treatment, factors to predict relapses, and long-term outcomes of eculizumab discontinuation.

## Graphical abstract

**Figure:**
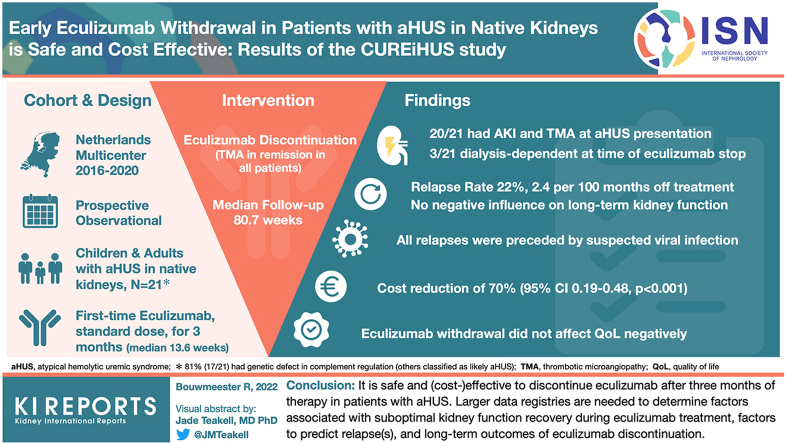

See Commentary on Page 4

In 2012, the complement C5-inhibitor eculizumab was approved for patients with aHUS. aHUS is a rare and severe form of TMA characterized by vascular occlusion of glomerular capillaries because of thrombus formation, leading to thrombocytopenia, microangiopathic hemolytic anemia, and acute kidney injury (AKI).^1^ In 50% to 70% of patients with aHUS, predisposing genetic mutations are found in complement (regulatory) proteins, leading to dysregulation and overactivation of the alternative pathway of the complement system.^2^^,^^3^ In the pre-eculizumab era, plasma therapy (PT) was the cornerstone of treatment. Outcome of this treatment was poor, with up to 10% mortality in the acute phase and 50% of patients progressing to kidney failure.^4^, ^5^, ^6^ Introduction of eculizumab has remarkably improved morbidity and mortality for patients with aHUS.

According to the summary of product characteristics of eculizumab, published by the European Medicines Agency, treatment consists of weekly eculizumab infusions during the induction phase (up to 4 weeks) followed by biweekly infusions as maintenance therapy.^7^ Lifelong therapy was advocated because of a potential risk of relapse and chronic kidney disease after eculizumab withdrawal. However, discontinuation of PT has been historically feasible in a substantial number of patients with aHUS.^4^, ^5^, ^6^, ^7^, ^8^ Therefore and in view of the extremely high costs, prominent risk of meningococcal infection, and unknown side effects of long-term eculizumab treatment, the need for continued eculizumab treatment has been a matter of debate.^9^ The costs of lifelong treatment easily exceed the thresholds for willingness to pay per quality-adjusted life-year. These costs might impede reimbursement policies and consequently patient health benefits in countries with collective health care insurance, such as the Netherlands.

Recent studies suggested that eculizumab discontinuation and tapering is feasible and probably cost-effective.^10^, ^11^, ^12^, ^13^, ^14^ However, these studies were mostly retrospective, included patients with an uncertain diagnosis, and were likely biased toward inclusion of low-risk patients.^15^ In addition, there was a great intrastudy and interstudy variation in eculizumab treatment duration, leaving the optimal timing of withdrawal unknown. Furthermore, follow-up duration was relatively short in these studies.^10^^,^^14^

Therefore, we conducted a 4-year, prospective, national, multicentre study to assess the safety and cost consequences of a restrictive eculizumab treatment strategy in an unselected cohort of patients with aHUS in native kidneys.

## Methods

This prospective observational study, CUREiHUS (NTR5988), was conducted from January 1, 2016, to October 1, 2020, in all university medical centers in the Netherlands. Pediatric and adult patients with suspected aHUS (first episode or relapse) and first-time eculizumab treatment were eligible for inclusion.

Patients with confirmed thrombotic thrombocytopenic purpura (ADAMTS13 activity level <10%) or secondary TMA occurring as a complication of pre-existing disease or precipitated by various conditions (such as shiga-toxin–producing *Escherichia coli* HUS, *Streptococcus pneumoniae* HUS, malignancy [i.e., monoclonal gammopathy], autoimmune disease [i.e., antiphospholipid syndrome], drugs, malignant hypertension, pregnancy, and metabolism disorders [i.e., cobalamin C deficiency]) were excluded.

After starting therapy, genomic analysis was performed to screen for variants in complement factor H (CFH), complement factor B, complement factor I, C3, membrane cofactor protein (MCP/CD46), CFH-related proteins 1–5, diacylglycerol kinase-ε, and thrombomodulin. Multiplex ligation–dependent probe amplification was performed to detect genomic rearrangements in the CFH/CFH-related protein region. In addition, homozygous presence of at-risk *CFH-H3* and *MCPggaac* haplotypes was evaluated.^16^^,^^17^ Genetic variants were classified as (likely) pathogenic, variant of uncertain significance, or (likely) benign, compliant with the American College of Medical Genetics and Genomics guidelines.^18^ In patients older than 30 years, detection of autoantibodies against CFH was performed using an in-house enzyme-linked immunosorbent assay.^19^

To reassess aHUS diagnosis before final data analysis, all patients suspected of having aHUS were categorized in retrospect as definite aHUS, possible aHUS, or secondary TMA after extensive evaluation of laboratory values and histology during the TMA episode and results of genetic analysis (Supplementary Method S1). Patients with definite or possible aHUS in native kidneys were considered eligible for further analysis. Patients with suspected aHUS who did not receive eculizumab, patients with suspected aHUS in transplanted kidney, and patients with suspected aHUS who were on chronic kidney replacement therapy were excluded from analysis.

### Eculizumab Treatment

All patients were treated prospectively according to a restrictive eculizumab protocol since January 2016. The treatment algorithm of the Dutch guideline for the treatment of patients with aHUS in native kidney is illustrated in Supplementary Figure S1. The rationale and the details of the protocol, including eculizumab interval elongation and discontinuation, have been described previously.^20^

In adults without a medical history of TMA, initial treatment consisted of PT for a period of 4 days to allow proper diagnostics to exclude other causes of TMA. If no other cause of TMA was found and the patient did not respond to PT or was PT dependent, eculizumab treatment was started. Eculizumab was started immediately in children and in patients with a disease recurrence (and prior PT dependency or resistance or need for eculizumab). Eculizumab was given at the standard dose, as advised by the European Medicines Agency and the US Food and Drug Administration for a period of 3 months.^21^ After 3 months, treatment was stopped or continued at elongated dose intervals, depending on, among others, normalization of TMA parameters, stabilization of kidney function, blood pressure control, age, medical history, and patient and physician preferences.^20^ Eculizumab discontinuation was independent of underlying complement abnormalities.

### Follow-up

Regular follow-up at the outpatient clinic was coordinated by the treating physician. At each visit, TMA parameters, kidney function, and blood pressure were evaluated. During eculizumab treatment, eculizumab trough levels and/or the classical complement pathway activity (CH50) were measured. Other complement activation markers (i.e., C3d, sC5b9) were assessed in the acute phase and in case of suspected relapse. Laboratory assays have been previously described.^22^ After eculizumab discontinuation, all patients were instructed to promptly contact the hospital in case of signs of infection, fever, generalized malaise, hematuria and/or oliguria, edema, or any other sign of aHUS relapse. On indication, patients performed home blood pressure measurement and/or home urine dipsticks to screen for hypertension, and albuminuria or hemoglobinuria, respectively. All adverse events, including serious ones, were registered during follow-up.

### Relapse

We evaluated all relapses after interval elongation (≥2 weeks interval between eculizumab administrations) or withdrawal of eculizumab. A relapse was defined as the need for intensifying eculizumab therapy (either re-start of therapy or shortening of the dosing interval) during an event of AKI in combination with laboratory evidence of TMA and/or histologic evidence of acute TMA (details in Supplementary Method S2). The decision to intensify eculizumab therapy was made by the treating physician. Only patients with a (partial) recovery of kidney function during eculizumab therapy were considered at risk for relapse. In addition, in patients with a rise in sCr between eculizumab discontinuation and last follow-up but without the need for intensivation of eculizumab therapy, TMA parameters were evaluated in detail. In patients with stable sCr, clinically relevant relapses were considered absent. Furthermore, we evaluated episodes of hematological TMA in the absence of AKI after eculizumab discontinuation. These events were defined as ≥2 laboratory parameters suspect for TMA (thrombocytopenia [platelet count <150 × 10^9^/l]), lactate dehydrogenase (LDH) above the upper limit of normal (>250 U/l), and low or undetectable haptoglobin (<0.3 mg/l).

### Cost-Consequence Analysis

An extensive cost-consequence analysis was performed for all patients. The direct medical costs at patient level were determined at presentation, during follow-up, and during relapse. Among others, costs for eculizumab administrations, dialysis, aHUS genetic evaluation, kidney biopsy, laboratory assays, and comedication were included. The costs of our cohort were compared with a fictive scenario in which all patients would receive eculizumab following the standard European Medicines Agency scheme. To evaluate the effect of eculizumab discontinuation on health-related quality of life and measure health-care–related productivity losses, the EuroQol-5D questionnaire and Medical Technology Assessment Productivity Costs Questionnaire were used, respectively. Detailed methods are provided in Supplementary Method S3.

### Statistical Analysis

Clinical characteristics, including received eculizumab therapy and correlation between relapse and outcome, were descriptively expressed. Laboratory values were presented as quantitative data. For continuous variables, values were expressed using median, and range (minimum–maximum) was used. Estimated glomerular filtration rate (eGFR) was estimated using the Chronic Kidney Disease-Epidemiology Collaboration formula in adults and the revised bedside Schwartz formula in children.^23^

Costs per patient were calculated as mean costs and presented with minimum and maximum costs. Utility was calculated per patient following the Dutch EQ-5D tariff.^24^ Total costs (per patient per unit of time) were presented descriptively. To determine differences in cost between groups, generalized linear models were used with a gamma distribution (to account for skewness in the data) and an identity link or loglink. Because of the small sample size, EQ-5D utilities of patients with and without disease recurrence were presented for individual patients. Statistical analyses were performed using IBM SPSS Statistics (V.25.0: IBM) and figures were drawn using GraphPad Prism (V5.03) or Microsoft Office Excel (V.2016).

### Data Collection

Ethical approval was obtained in the Netherlands from the Medical Research Ethics Committee of Oost-Nederland (registration number of the CUREiHUS study: NL52817.091.15). This trial is registered at the Dutch Trial Registry NTR5988. Informed consent was obtained from all patients before inclusion in the study. Start of prospective inclusion was dependent on local ethical approval, which differed by center. Patients who started with eculizumab before local study initiation (but after January 1, 2016) were treated according to the guideline and followed by their treating physician with evaluation of routine clinical data. After ethical approval and obtaining patient informed consent, retrospective data were collected and entered into the database, and patients were prospectively followed.

## Results

**Figure 1:**
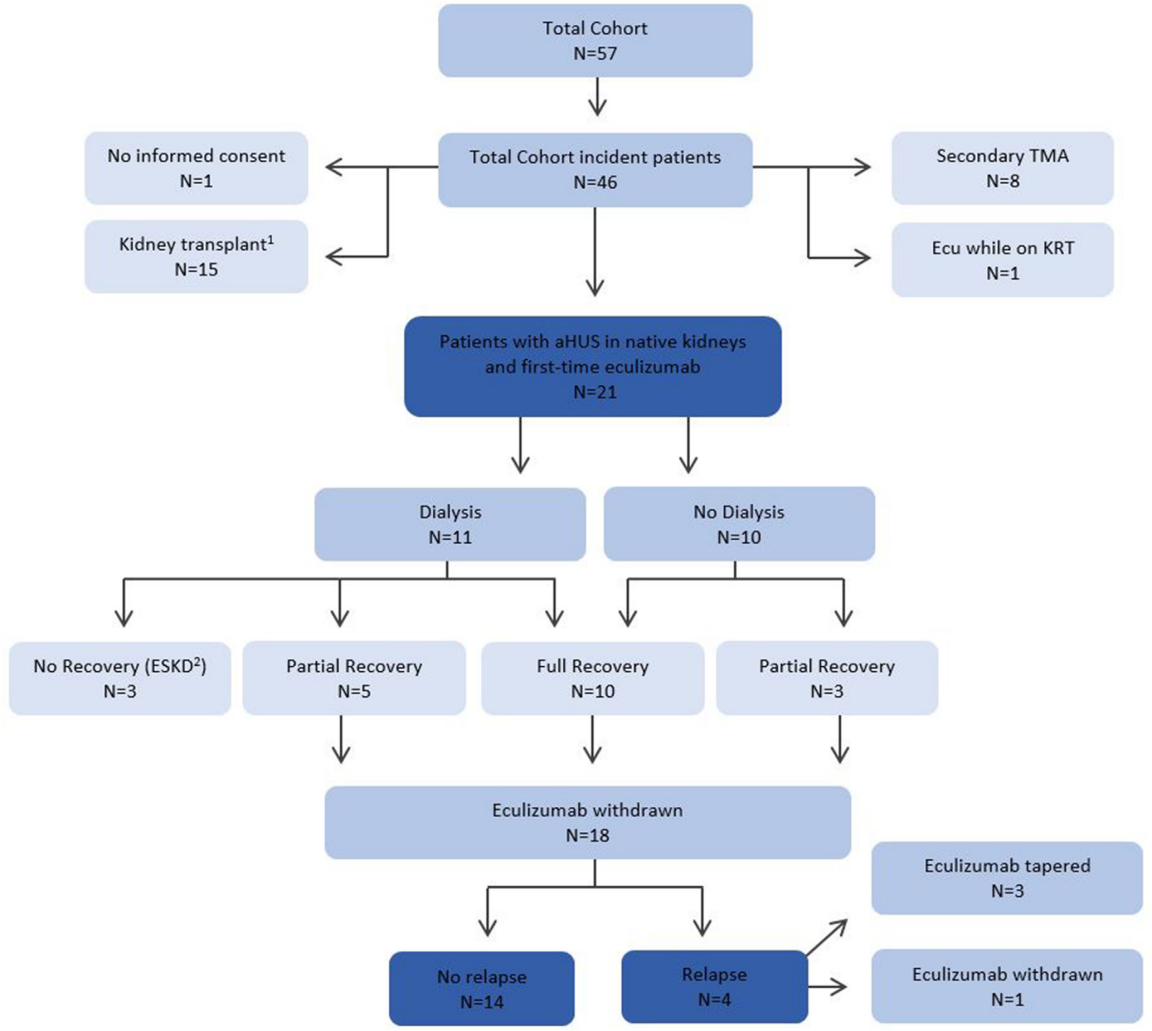
Flowchart of eculizumab therapy and outcomes in patients with suspected aHUS in native kidneys. In addition to the excluded patients, this figure shows our cohort, their treatment with dialysis and eculizumab, and outcomes (relapse vs no relapse). ^1^These patients had a suspected aHUS recurrence after kidney transplantation. ^2^In these patients, kidney function did not recover. These patients did discontinue eculizumab and were not included in the analysis of relapse. Of note, in none of these patients eculizumab was restarted during follow-up on hemodialysis. aHUS, atypical hemolytic uremic syndrome; Ecu, eculizumab; ESKD, end-stage kidney disease (kidney failure); KRT, kidney replacement therapy; TMA, thrombotic microangiopathy.

Overall, eculizumab was initiated for suspected aHUS in 46 patients. The flowchart of patient inclusion in depicted in Figure 1. In 8 patients, a diagnosis of secondary TMA was made in retrospect, and these patients were excluded from further analysis. These patients received 3 (1–8) doses of eculizumab, which was stopped after establishing a diagnosis. One patient was known with kidney failure, and 1 patient did not sign informed consent. In 15 patients, eculizumab was initiated for suspected aHUS recurrence after kidney transplantation. These patients were analyzed separately.

Therefore, this analysis included 21 patients with aHUS (14 adults and 7 children), who were treated with eculizumab. Three patients had a previous aHUS episode (median duration between first presentation and inclusion in the study was 14.3 years [3.4–14.6 years]), whereas 18 patients had new-onset aHUS. Median age of aHUS onset in all patients was 26.3 years (0.3–78.5 years). Characteristics of the patients are shown in Table 1.

**Table 1:** Characteristics of patients with aHUS in native kidneys

Characteristics	Total, *N* = 21	Adults, *n* = 14	Children, *n* = 7
Sex			
Female	11 (52%)	8 (57.1%)	3 (42.9%)
Male	10 (48%)	6 (42.9%)	4 (57.1%)
Median age (range), yr	31.8 (0.3–78.5)	41.5 (24.9–78.5)	5.9 (0.3–13.1)
Patients with a complement genetic variant^a^			
*CFH*^b^	16 (76.2%)	12 (85.7%)	4 (57.1%)
*MCP*	8^c^ (42.9%)	7 (50%)	1 (14.3%)
*CFI*	3 (14.3%)	1 (7.1%)	2 (28.6%)
*C3*	2 (9.5%)	1 (7.1%)	1 (14.3%)
*CFB*	6 (28.6%)	5 (35.7%)	1 (14.3%)
Patients with >1 complement genetic variants^a^	0 (0%)	0 (0%)	0 (0%)
Antifactor H antibodies	3 (19.1%)	2 (14.3%)	1 (14.3%)
Patients with a complement genetic variant^a^ and:	2 (9.5%)	0 (0%)	2 (28.6%)
MCP*ggaac* haplotype homozygosity	3 (14.3%)	2 (14.3%)	1 (14.3%)
CFH-H3 haplotype homozygosity	1 (4.8%)	1 (7.1%)	0 (0%)
Medical history			
Previous aHUS episode^d^	3 (14.3%)	3 (21.4%)	0 (0%)
aHUS family history^e^	6 (28.6%)	5 (35.7%)	1 (14.3%)
CKD^f^/hypertension	3 (14.3%)	3 (21.4%)	0 (0%)
aHUS potential triggering event			
Suspected viral infection	11 (52.4%)	5 (35.7%)	6 (85.7%)
Influenza infection^g^	4 (19.0%)	1 (7.1%)	3 (42.9%)
Other viral infection	7 (33.3%)	4 (28.6%)	3 (42.9%)
Bacterial infection	3 (14.3%)	3 (21.4%)	0 (0%)
Pregnancy related	3 (14.3%)	3 (21.4%)	–
Postsurgery	1 (4.8%)	1 (7.1%)	0 (0%)
Vaccination^h^	1 (4.8%)	0 (0%)	1 (14.3%)
Uncontrolled hypertension^i^	2 (9.5%)	2 (14.3%)	0 (0%)
Laboratory parameters at first day of TMA^j^			
Serum creatinine (μmol/l)	200 (42–2792)	312.5 (86–2792)	119 (42–473)
eGFR (ml/min per 1.73 m^2^)	22 (1–80)	17.5 (1–80)	34 (11–56)
UPCR (g/10 mmol)^k^	7.08 (0.12–19.60)	4.34 (0.12–11.25)	15.82 (8.51–19.60)
Hypertension, n (%)	12 (66.7%)	8 (72.7%)	4 (57.1%)
Thrombocytes (×10^9^/l)	57 (3–186)	87.5 (14–186)	51 (3–92)
Hemoglobin (mmol/l)	5.1 (3.2–8.6)	4.85 (3.2–8.6)	6.1 (3.4–7.3)
LDH (U/l)^l^	1604 (299–5414)	1029 (299–5414)	2920 (2552–3538)
Haptoglobin (g/l)^m^	<0.3 (<0.3–1.0)	<0.3 (<0.3–1.0)	<0.3 (<0.3)
Bilirubin (μmol/l)^l^	28 (5–120)	23 (3–120)	30 (23–97)
ALAT (U/l)^l^	24.5 (7–321)	22 (7–321)	59 (12–186)
ASAT (U/l)^n^	71 (17–970)	59 (17–970)	123 (108–288)

aHUS, atypical hemolytic uremic syndrome; ALAT, alanine aminotransferase; ASAT, aspartate aminotransferase; CKD, chronic kidney disease; DPTP, diphtheria-pertussis-tetanus-polio; eGFR, estimated glomerular filtration rate; ESKD, end-stage kidney disease; LDH, lactate dehydrogenase; TMA, thrombotic microangiopathy; UPCR, urinary protein-to-creatinine ratio.

a Including complement gene class III rare variants of unknown significance, class IV variants (likely pathogenic), and class V variants (pathogenic).

b Including *n* = 3 hybrid CFH proteins. Single CFHR variants are not included.

c *n* = 7 with a CFH genetic variant. One adult patient had 2 CFH variants.

d Only aHUS episodes that were not treated with eculizumab.

e Family history was defined as at least 1 family member with a (officially diagnosed) medical history of aHUS. Unaffected family members with only a (likely) pathogenic variation (carriers) were not included.

f CKD defined as eGFR <60 ml/min per 1.73 m^2^. Of all patients with a previous episode of TMA/aHUS, CKD was diagnosed in 2 of 3 patients (67%) before the start of the CUREiHUS study. The remaining patient had CKD because of hydronephrosis and uncontrolled hypertension. Patient(s) with pre-existing ESKD were excluded.

g Influenza A, *n* = 3. Influenza B, *n* = 1.

h 3 days post DPTP vaccination.

i No trigger was identified other than (malignant) acceleration of pre-existent hypertension. Both patients had a (likely) pathogenic complement genetic variant.

j First day of suspected TMA diagnosis. Of all parameters, median values (ranges) are shown.

k *n* = 11 missing.

l *n* = 3 missing. LDH upper limit of normal: 250 U/L.

m *n* = 7 missing.

n *n* = 5 missing.

At presentation, ≥2 abnormal TMA parameters were present in 17 patients. Within 6 days after presentation, ≥2 TMA parameters were measured and abnormal in all 21 patients. Median lowest platelet counts (50 × 10^9^/l, 3–115) and highest lactate dehydrogenase levels (2270 U/l, 248–5586) were found on day 3 (0–16) and 0 respectively. To exclude thrombotic thrombocytopenic purpura, ADAMTS13 activity levels were determined in all patients (*n* = 19) with platelet counts <150 × 10^9^/l on the first day of TMA. All but 1 patient had AKI at presentation (≤7 days since the start of TMA), 4 of 20 patients RIFLE (risk, injury, failure, loss of kidney function, and end-stage kidney disease) criteria stage 2 (injury, increase in sCr of ≥2 times baseline), and 16 of 20 patients stage 3 (failure, increase in sCr of ≥3 times baseline). One patient with a medical history of aHUS presented with a gradual increase in sCr during early pregnancy. Median highest sCr level (417 μmol/l, 109–4792) was found at TMA day 5 (0–24), and 17 of 21 patients developed acute kidney disease with at least a doubling in sCr (day 7–90).

In 17 patients (81%), a defect in complement regulation was found by either a proven or likely pathogenic mutation or a rare variant of uncertain pathogenicity (variant of uncertain significance) in one of the complement regulatory genes or by antibodies against factor H. These patients presented after an evident triggering event and could be classified as definite aHUS (AKI and TMA) (Supplementary Tables S1 and S2). Four patients (14%) with either no or a likely benign variant and without a family history of aHUS were classified as possible aHUS.

### Initial Treatment

Eleven patients (52%) needed kidney replacement therapy (dialysis) (Supplementary Table S3). In line with the treatment guideline, all adult patients were treated with PT before the start of eculizumab for a median duration of 4 days (1–50 days). In contrast, eculizumab was started as primary, first-line therapy, in 6 of 7 children. In 1 child, 1 session of PT was given in await of eculizumab. Both patients with CFH autoantibodies were adequately treated with immunosuppression (mycophenolate mofetil and prednisone).

### Eculizumab Treatment and Discontinuation

**Figure 2:**
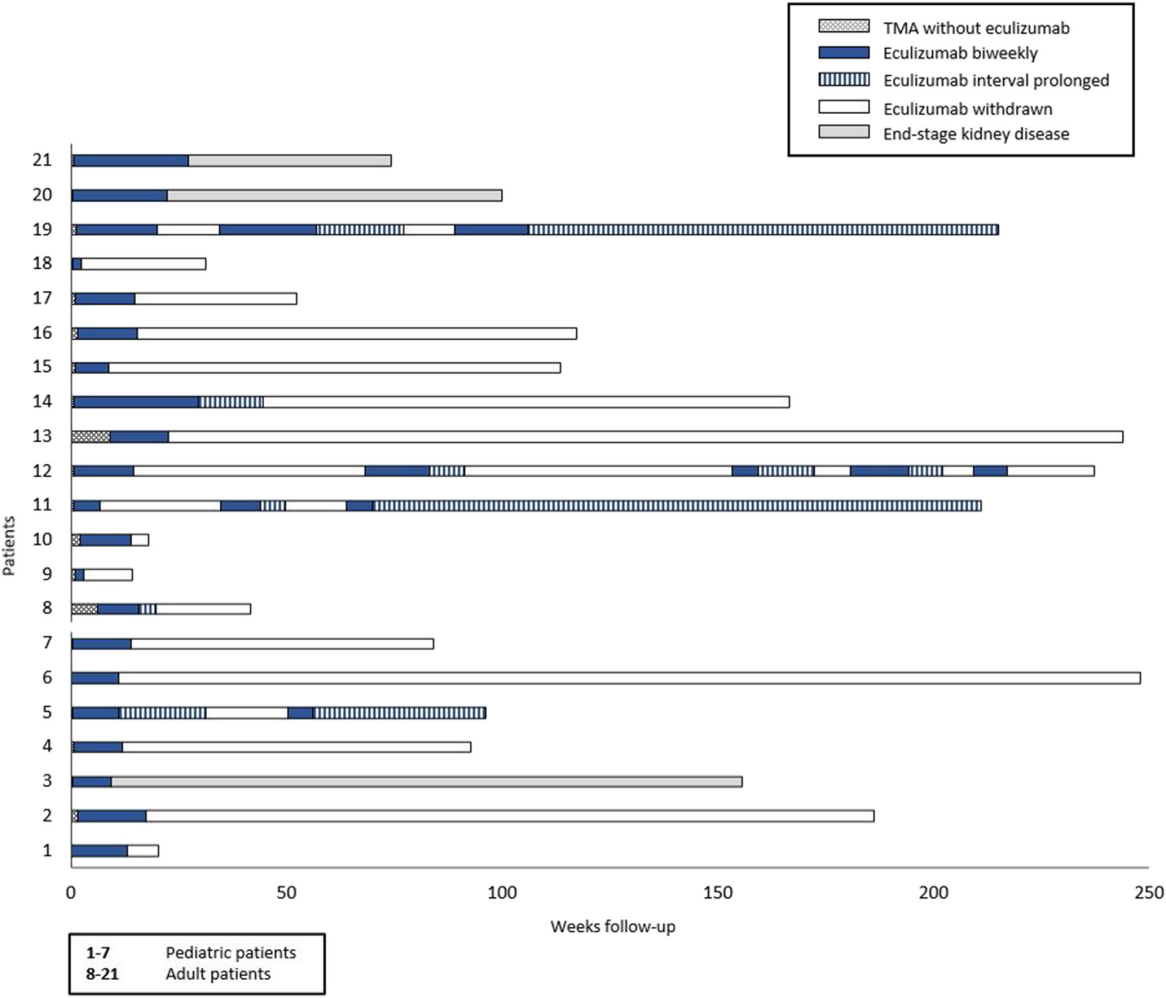
Schematic overview of follow-up and eculizumab treatment in patients with aHUS in their native kidneys. This figure provides an overview of eculizumab treatment over time in all patients. aHUS, atypical hemolytic uremic syndrome; TMA, thrombotic microangiopathy.

Eculizumab was started after a median of 3 (1–11) days and 6 (2–63) days after start of TMA in patients treated without and with PT, respectively. Post aut propter eculizumab therapy, TMA signs (hemolysis and thrombocytopenia) disappeared in all patients (Supplementary Table S4). Eculizumab was withdrawn in all patients after a median of 13.6 (2.1–43.9) weeks. In 3 patients, eculizumab interval was extended before discontinuation (due to ongoing pregnancy, age <6 years, and recurrent infections), while maintaining full complement inhibition (CH50 <10%) in 2 patients. In the remaining patient, eculizumab was administered on a 4-weekly interval only once before discontinuation (CH50 unknown). Three patients, in whom therapy was not extended, were treated with eculizumab for more than 16 weeks. Two patients had kidney failure and an ongoing, gradual recovery in kidney function. Follow-up is illustrated in Figure 2.

At the time of eculizumab discontinuation, TMA was in remission in all patients. Kidney function recovery was negligible in 3 patients (2 adults and 1 child), who remained on dialysis. Kidney function was improved in 18 patients, although full recovery (to normal or baseline eGFR) was noted in only 10 patients (4 adults and 6 children) with a median duration to full recovery of 27.5 days (6–67). Incomplete recovery was notable in 8 adult patients. We could not determine factors associated with (partial) recovery in kidney function in adult patients. In 6 of 8 patients, kidney function recovery was stabilized (<10% difference in sCr values) at the time of discontinuation. However, in 2 patients, it cannot be excluded that kidney function could have improved to some further extent if eculizumab had been continued (details in Supplementary Results).

### Relapse

**Figure 3:**
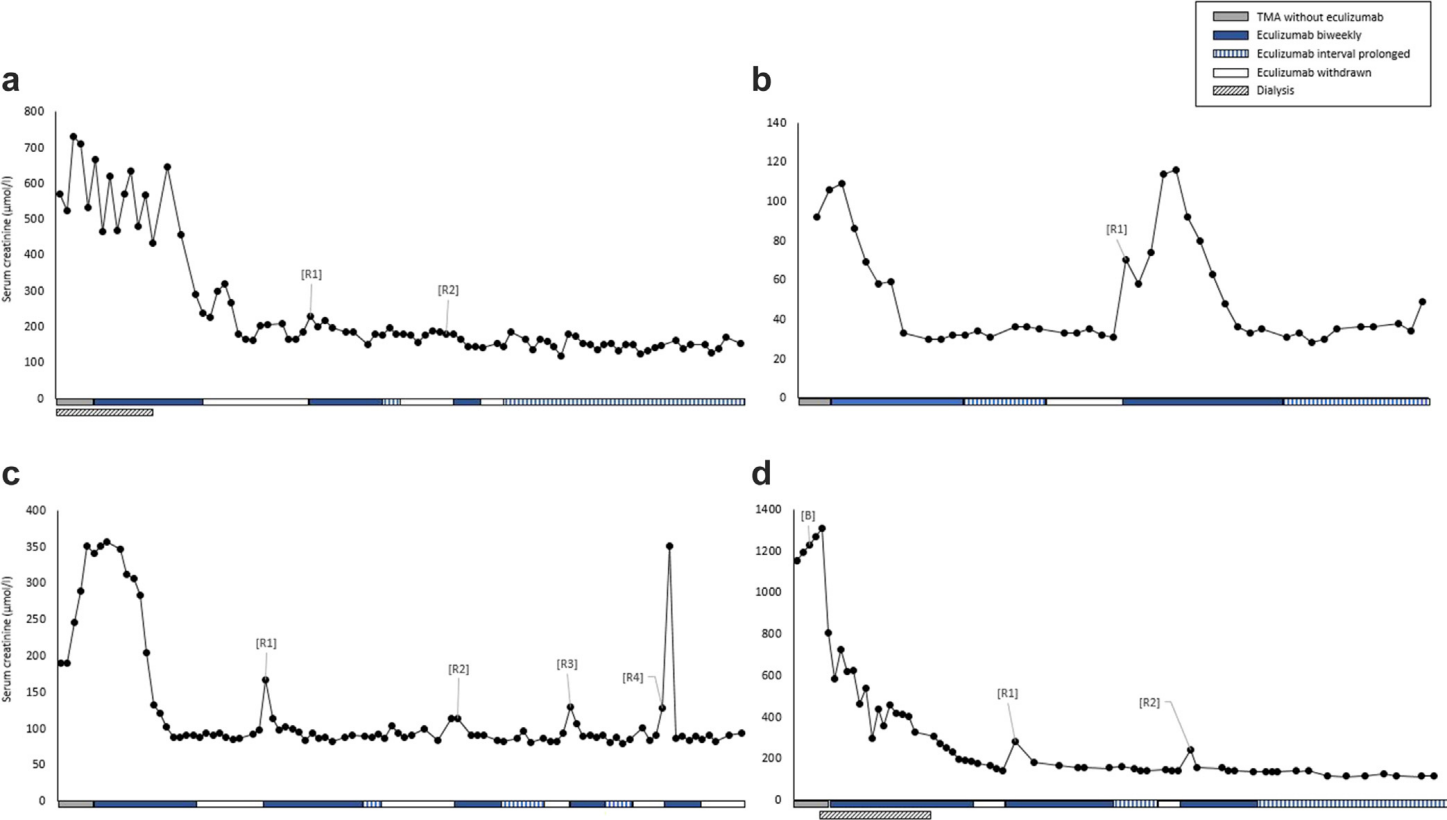
Serum creatinine (μmol/l) concentrations over time in the 4 patients with relapsing aHUS. This figure shows the serum creatinine (μmol/l) concentrations over time of the 4 relapsing patients. The letters correspond to the following patient numbers: [A] 11, [B] 5, [C] 12, [D] 19. aHUS, atypical hemolytic uremic syndrome; B, biopsy; PT, plasma therapy; R, relapse.

A first relapse occurred in 4 patients (3 adults and 1 child), respectively, 14.3, 19.0, 20.0, and 53.6 weeks (median 19.5 weeks) after eculizumab withdrawal. All relapses were preceded by complaints compatible with viral infections, which made patients, as instructed by the physician, actively seek medical attention. As a result, all relapses were detected early, and dialysis was not needed. In addition, extrarenal manifestations of TMA were absent in all relapses. Renewed treatment (≤1 day after start of TMA) with eculizumab resulted in full recovery of kidney function and hematological TMA parameters (Figure 3, Supplementary Tables S5 and S6). Renewed withdrawal in 3 patients was associated with additional relapses, all manageable (details are provided in Supplementary Table S5 and Supplementary Results). During follow-up after eculizumab discontinuation, signs of TMA were observed in 1 patient (Supplementary Results). This episode resolved spontaneously, without the need for eculizumab reinitiation.

Patients who remained on dialysis were not considered at risk of relapse. Therefore, during follow-up after eculizumab discontinuation (80.7 weeks [0.0–236.9]), the clinical relapse rate in this cohort was 22% (4/18). Overall, the calculated relapse rate was 2.4 per 100 months of observation off treatment. Of all patients with ≥1 year of follow-up after eculizumab discontinuation (12 of 18), the relapse rate was 33%. There were no clinical differences between relapsing and nonrelapsing patients (Supplementary Table S6). Notably, a likely pathogenic complement variant was identified in all relapsing patients. In addition, relapse rates for patients with and without a complement genetic variant were 25% (4/16) and 0% (0/2), respectively. Despite the limited number of relapsing patients, relapses did not seem to influence long-term kidney function negatively (Figure 3; Supplementary Tables S5 and S6). The paucity of relapsing patients does not allow identification of biomarkers for the prediction of relapse. However, in 1 patient, increasing sCr or urinary protein-to-creatinine ratio at the latest outpatient visit (respectively 22 and 28 days) before 2 relapses might have been predictive of relapse.

### Long-term Outcomes

From eculizumab discontinuation to last follow-up, changes in kidney function (≥15% increase in sCr or ≤5 ml/min per 1.73 m^2^ decrease in eGFR) were noted in 2 nonrelapsing patients with a diagnosis of possible aHUS and partial recovery in kidney function after eculizumab treatment. One patient, a 78-year-old man, developed heart failure and cardiac arrhythmias, treated with amiodarone and furosemide, which likely caused the increase of sCr from 122 to 157 μmol/l, yet remained stable thereafter. There were no signs of TMA during the period of kidney function deterioration. Another patient, a 55-year-old woman, had an episode of self-limiting TMA and AKI without the need for reinitiation of eculizumab (details in Supplementary Results). At last follow-up of the study (32 weeks after this event), kidney function recovery was still ongoing, and there we no signs of irreversible kidney damage. Kidney function recovered to baseline 19 weeks later.

Overall, there was no evidence of additional, unexplained kidney injury after eculizumab discontinuation and/or relapse (Supplementary Tables S4 and S6). In addition, proteinuria decreased after therapy, and proteinuria at end of follow-up was limited (0.15 g/d).

During follow-up, 10 serious adverse events other than (related to the) aHUS relapses were registered. Eight serious adverse events were infections, of whom 5 required hospitalization. Six viral infections occurred after eculizumab discontinuation, and patients were adequately evaluated on aHUS disease activity parameters. Side effects of eculizumab occurred twice; a meningococcal infection (despite MenACWY-B vaccination) and (potential) eculizumab-associated hair loss. In addition, 1 bacterial infection could be related to prednisone/mycophenolate mofetil usage in a patient with CFH autoantibodies. One patient with kidney failure successfully received kidney transplantation during the study without prophylactic eculizumab treatment.

### Cost-Consequence Analysis

#### Medical Costs

To determine the exact costs of our restrictive regime, we included all medical costs besides mere eculizumab administrations. Total costs (mean, minimum–maximum) during the study period per patient were €288,778 (€58,013–€1,098,698) and per month of follow-up was €11,537 (€1712–€34,042). Costs of our cohort were only 30% (95% confidence interval 0.19–0.48, *P* < 0.001) of costs in the fictive scenario that all our patients would have received eculizumab following the standard regimen. The mean costs per patient per month of this fictive cohort would be €34,529 (€10,981–€48,447). Mean costs per month of patients with disease recurrence were €16,931 (€8900–€22,245) compared with patients without disease recurrence with mean (minimum–maximum) costs per month of €10,269 (€1720–€34,042).

On average, eculizumab administrations (mean costs €251,826 [€21,923–€1,071,487], including costs of outpatient clinic and laboratory evaluation) accounted for 87% of the total costs per patient. Most of the remaining costs were expenses made at presentation, including intensive care unit admission, hospital admission, period of dialysis, and laboratory evaluation. Of note, comedication besides eculizumab was only accountable for 1% of the total expenses. Travel costs and productivity losses were limited, with costs of €634 per patient during the study period and losses of €655 (€0–€3263) per patient per month, respectively.

### Quality of Life

Of the 21 patients in our study, 13 (11 adults, 2 children) responded to the questionnaire measuring quality-adjusted life-year. Of these patients, 2 experienced disease recurrence during follow-up. In 1 patient, the utility remained 1 (perfect score) during 2 consecutive relapses, in the second patient, utility dropped shortly from 1 to 0.8 at the time of the first relapse and returned to 1 after eculizumab withdrawal. During the 3 following relapses in this patient, utility remained 1.

In the total cohort, utility fluctuated strongly probably because of the highly heterogeneous patient population and various comorbidities. However, eculizumab withdrawal appeared not to influence quality of life negatively because utility scores remained stable or even increased after eculizumab withdrawal (Supplementary Figure S2).

## Discussion

This is the first prospective, observational study demonstrating the safety of unbiased, controlled, and clinician-directed eculizumab discontinuation in adult and pediatric patients with aHUS in native kidneys. It is evident that the study included well-characterized patients, representative of typical aHUS. Although approval for eculizumab was based on clinical reasoning and genetic analysis was not yet available, the number of patients with ≥1 complement genetic variant in our cohort was relatively high (71%). In addition, 17 of 21 patients (81%) could be classified as definite aHUS according to our proposed classification system (Supplementary Method S1), and ≥2 parameters of TMA and AKI were present in all patients within 6 days since presentation.

During follow-up after discontinuation of eculizumab (80.7 weeks [0.0–236.9]), the relapse rate was 22%. A first relapse (all defined by the presence of both TMA and AKI parameters) occurred after a median duration of 19.5 (14.3–53.6) weeks, and suspected viral infections were triggering events of all relapses. Most importantly, chronic sequelae (clinically relevant increase in sCr, proteinuria, and/or hypertension) were not observed in relapsing patients, who all received renewed treatment with eculizumab. In addition, after eculizumab discontinuation, no additional kidney damage was found in all 8 patients with chronic kidney disease, including 2 patients who relapsed twice. Our data extend and strengthen the findings of other published aHUS cohorts. In these cohorts, treatment cessation was somewhat biased, and/or transparency regarding clinical parameters that might have affected patient selection was insufficient. Nevertheless, these studies reported the following: (i) relapse rates ranging from 20% to 32%, (ii) that most relapses would occur within a year after eculizumab discontinuation, and (iii) that discontinuation was not associated with additional or progressive kidney damage in most patients.^10^^,^^11^^,^^13^^,^^14^^,^^25^, ^26^, ^27^, ^28^

The median time of eculizumab treatment (range 2.4–19.6 months) varies greatly between cohorts, clearly illustrating the difficulties in clinical decision making.^10^^,^^11^^,^^13^^,^^14^^,^^28^^,^^29^ Yet, this study demonstrated the feasibility of early (median 3 months) withdrawal. Hematological TMA parameters were in remission, and kidney function was stabilized in the majority (19 of 21) of patients at time of discontinuation. In addition, median duration to full kidney function recovery was only 27.5 days (6–67), and in 6 patients, eculizumab could even be successfully withdrawn after <12 weeks of treatment, advocating the possibility of an even more personalized treatment regimen. Of note, kidney function recovery was partial and negligible in 8 adult and 3 (2 adult/1 pediatric) patients, respectively. All but 1 pediatric patient showed full recovery in kidney function. In adult patients, we could not determine factors associated with partial recovery. However, we suggest that diagnostic delays and severe kidney injury at presentation contribute to incomplete recovery.^30^

Furthermore, to our knowledge, this is the first study to provide an extensive cost-consequence analysis of eculizumab discontinuation. It confirms that eculizumab administration is a cost driver because it accounted for 87% of the total costs per patient in our cohort. Withdrawal resulted in a 70% reduction in costs, compared with the hypothetic scenario that our patients would have received eculizumab following a continued, biweekly regime. In addition to this enormous cost reduction, eculizumab discontinuation did not seem to negatively influence quality of life.

The safety of eculizumab withdrawal was questioned in one study that reported a 50% reinitiation rate after eculizumab withdrawal and a decreasing kidney function over time after discontinuation.^29^ This study included 93 patients, who participated in one of the eculizumab trials and of whom follow-up data were available. In 42 patients, eculizumab was discontinued after a treatment period of 19.6 (0.2–86.9) months. There is no information on patients selected for withdrawal. Although 21 patients (50%) reinitiated treatment with eculizumab, the reason for renewed therapy was TMA in only 11 patients (TMA relapse rate of 26%). The authors concluded that eGFR remained stable in patients who continued treatment, whereas there was a trend to decline in patients who discontinued treatment. This conclusion can be debated because median eGFR was 59.5 ml/min per 1.73 m^2^ in the eculizumab continuation group at last follow-up. In the discontinuation group, median eGFR was 92.3 ml/min per 1.73 m^2^ at discontinuation and 75.6 ml/min per 1.73 m^2^ at last follow-up. This apparent decrease in eGFR could simply reflect regression to the mean. It is also unknown if this eGFR decrease was related to recurrent TMA events. Furthermore, it is not stated if eculizumab was reinitiated promptly within days after TMA recurrence. The time from the most recent TMA episode to the start of eculizumab was on average 6.6 months, suggesting some delay. Therefore, these results should be interpreted with caution.

Our study also had some limitations. Start of prospective inclusion was dependent on local ethical approval, which differed by study site. Moreover, a longer follow-up duration is needed to determine late outcomes. Our study included 21 patients with aHUS. The low sample size and the paucity of relapsing patients did not allow assessment of clinically relevant predictors of relapse. It is notable that none of our patients without a complement variant developed a relapse over time. This strengthens the evidence of an absent complement gene mutation currently being the only apparent negative predictor of relapse (negative predictive value 90%). In other cohorts, remaining clinical factors yielded insufficient positive predictive values, including CFH or MCP variants, age <18 years, previous TMA episode(s), and serum complement assays.^10^, ^11^, ^12^, ^13^, ^14^^,^^28^, ^29^, ^30^, ^31^, ^32^, ^33^, ^34^

It is questionable whether predictors of relapse should be the driving force in the management of eculizumab because, in our cohort, relapse appears not to be associated with negative kidney function outcome. Yet, patient adherence and collaboration are an absolute prerequisite for controlled, early eculizumab reinitiation during relapse and, thereby, kidney function preservation.^10^^,^^13^^,^^14^^,^^30^ At all times, both physician and patient should be aware of a potential aHUS relapse, especially during the first year after treatment cessation, potentially triggering events (mainly infections), and clinically relevant increases in sCr and/or hematuria and/or proteinuria and/or TMA.

In conclusion, this prospective study demonstrates the safety and cost-effectiveness of eculizumab withdrawal after 3 months of therapy in well-defined, pediatric and adult patients with aHUS in native kidneys. Our results emphasize the feasibility and, especially in countries with collective health care insurance, societal responsibility of controlled eculizumab withdrawal in patients with aHUS. In addition, we emphasize that continued eculizumab treatment is not without risks, and long-term outcomes of sustained complement inhibition are unknown. Clinician-directed discontinuation (including adherence to protocol and discontinuation conditional on stabilized kidney function), close monitoring, and patient collaboration seem to be a prerequisite for the safety of restrictive eculizumab management. In our study, suboptimal kidney function recovery during initial eculizumab treatment was the most important determinant for short-term and long-term outcomes. To improve outcomes in patients with aHUS, more research is needed to determine the influence of delayed hospital admission, diagnosis, start of PT, and/or eculizumab on initial kidney function recovery. Furthermore, larger data registries, preferably controlled and randomized trials, are needed to determine the long-term outcomes of eculizumab discontinuation and the actual distinctive value and clinical relevance of factors that predict relapses.

## Disclosure

JFMW, NCAJvdK, VG, MGK-V, AHMB, SPB, ED, JvdW, and ADvZ are members of the European Reference Network for Rare Kidney Diseases (ERKNet)-Project No 739532. JFMW is a member of the international advisory board of Alexion and also received a grant from Alexion. NCAJvdK has received a consultancy fee from Roche Pharmaceuticals and Novartis and is a subinvestigator in the APL2-C3G trial, Apellis. AHMB received a consultancy fee from Novartis and is a member of the DSMB Zoster-047 trial, GSK, and a subinvestigator in the Belatacept study, BMS. VG is a subinvestigator in the APL2-C3G trial, Apellis. All the other authors declared no competing interests.

## Supplementary Material

**Supplementary Methods.**

**Method S1**. aHUS diagnosis classification.

**Method S2**. aHUS relapse definition.

**Method S3**. Methods of cost-consequence analysis.

**Supplementary Results**

**Figure S1.** Treatment algorithm of the Dutch guideline in patients with (suspected) aHUS in native kidneys.

**Figure S2.** Individual utility scores over time.

**Table S1.** Genomic analysis of all patients.

**Table S2.** aHUS diagnosis classification of all patients.

**Table S3.** Noneculizumab treatment in patients with aHUS.

**Table S4.** Outcomes in patients with aHUS after initial eculizumab treatment, discontinuation, and last follow-up.

**Table S5.** Outcomes at eculizumab discontinuation and last follow-up in relapsing patients.

**Table S6.** Characteristics and outcomes of relapsing versus nonrelapsing patients (without ESKD).

**STROBE Checklist.**
