# Supplementary material for: Early Eculizumab Withdrawal in Patients With Atypical Hemolytic Uremic Syndrome in Native Kidneys Is Safe and Cost-Effective: Results of the CUREiHUS Study
Source: Kidney Int Rep. 2022 Oct 18;8(1):91–102. doi: 10.1016/j.ekir.2022.10.013 (PMC9832049; doi:10.1016/j.ekir.2022.10.013)
Supplement: Supplementary File (PDF) [file mmc1.pdf]

Supplementary Material and STROBE checklist

Supplementary Method S1 – aHUS diagnosis classification

For classification purposes laboratory values and histology during the TMA episodes were evaluated:

1] *TMA* was defined by ≥2 of the following criteria: thrombocytopenia (platelet count <150 x 10^9/L), lactate dehydrogenase (LDH) above the upper limit of normal (>250 U/l) and low/undetectable haptoglobin (<0.3 mg/L).

2] *Acute kidney injury* (AKI) was defined as an increase in serum creatinine of ≥26.53 µmol/L (0.3 mg/dl) in 48 hours or ≥1.5 times baseline in <7 days.

3] Kidney biopsies were reviewed for signs of acute and/or chronic TMA and other etiologies of kidney injury. Histological evidence of *active TMA* was defined as the presence of glomerular capillary, arterial and/or arteriolar thrombosis. *Chronic TMA* was defined as signs of glomerular ischemia, double contour formation of glomerular basement membranes, onion skin formation of vessels and/or endothelial swelling.

Classification

Definite aHUS was defined as:

A patient with a pathogenic or likely pathogenic variant or variant of uncertain significance (VUS) in genes encoding complement proteins or the presence of auto-antibodies against factor H. In addition, patients with AKI in combination with TMA *and* a positive family history of aHUS (first and/or second-degree relatives) were also classified as definite aHUS.

Possible aHUS was defined as:

Patients presenting with AKI in combination with laboratory evidence of TMA and/or histological evidence of acute TMA, but without a pathogenic or likely pathogenic complement mutation or variance of uncertain significance (VUS) and a negative family history for aHUS. A secondary cause of TMA was excluded.

Secondary TMA was defined as:

Patients who were initially diagnosed and treated (with eculizumab) as suspected aHUS. However, after careful analysis of all data, TMA could be attributed to a secondary cause (e.g. antiphospholipid syndrome, malignancy, pneumococcal infection, pregnancy related TMA, malignant hypertension, sepsis with DIC) or was caused by a genetic variant in non-complement genes (e.g. Cobalamin C deficiency, DGKE mutation, THBD mutation).

Supplementary Method S2 – aHUS relapse definition

Atypical HUS relapse was defined as:

The need for intensifying eculizumab therapy (either re-start of therapy or shortening of the dosing interval), during and/or after an event of AKI (RIFLE criteria stage ≥1 (an increase in serum creatinine of ≥26.53 µmol/L (0.3 mg/dl) in 48 hours or ≥1.5 times baseline in <7 days)) in combination with laboratory evidence of TMA and/or histological evidence of acute TMA. Hematological TMA was defined by ≥2 of the following criteria: thrombocytopenia (platelet count <150 x 10^9/L), lactate dehydrogenase (LDH) above the upper limit of normal (>250 U/l) and low/undetectable haptoglobin (<0.3 mg/L). Histological evidence of *active TMA* was defined as the presence of glomerular capillary, arterial and/or arteriolar thrombosis.

Supplementary Method S3 – Methods of cost-consequence analysis

At presentation, days of hospital admission (ICU and at medium care/ward in university hospital or general hospital), dialysis duration, laboratory analysis, genetic evaluation in light of aHUS workup, and (if applicable) kidney biopsy and erythrocyte transfusions were evaluated. Costs of laboratory assays performed per patient during the study period were obtained from published reference values for 2019 by the Dutch Healthcare Authority. At follow up, costs for all hospital visits (including emergency department (ED) visits including laboratory analysis were calculated. Costs of co-medication (besides eculizumab) were obtained from the guideline regarding Dutch healthcare research (www.medicijnkosten.nl accessed November 2020). Per patient, we reviewed all medication prescribed during the study period, yet incorporated only the expenses of vaccinations given in relation to eculizumab administration, antibiotics and medication with costs exceeding €5/day. Immunosuppressive medication, unrelated to kidney transplantation, were also included. In the Netherlands, reimbursement is based on fixed prices for a combination of diagnosis and treatment (DBC). Of note, costs of parameters not included in the DBC (i.e. intravenous immunoglobulin (IVIG)) were calculated separately. The expenses of erythropoietin (EPO) were incorporated in the DBC of dialysis. In case a patient received EPO without dialysis, costs were calculated.

Costs for eculizumab administrations were calculated at €4.384,53 ($4.964,76) per 300mg. Every time the patient received eculizumab on the ambulatory care unit, costs were collected for ambulatory care admission, laboratory assessment, and travel expenses made by the patient to visit the hospital. Using the route planner of ANWB (http://www.anwb.nl/verkeer/routeplanner), distance between patient accommodation and

the hospital was calculated. Costs of travel expenses were calculated with amount of kilometers traveled multiplied with €0,19 ($0,22) per kilometer.

Furthermore, costs of the fictive scenario that all patients would receive eculizumab following a standard regimen (maintenance phase with eculizumab every 2-3 weeks for the total study period per patient) were calculated. In this scenario we included medical healthcare expenses made per patient including baseline costs of presentation (similar to our cohort), biochemical evaluation, and eculizumab administrations including expenses of the ambulatory care unit with monthly biochemical evaluation. We assumed that no patient would experience disease recurrence in this 'best case' scenario.

Price index

Although the study ended in 2020, costs of 2019 were used in this study since the COVID-19 pandemic had a strong influence on our economy. To prevent our results being influenced by the pandemic, costs of 2019 were expected to reflect the normal situation. If costs were made in previous years, costs were modified using the change in consumer price index (CPI) from that year to 2019.

Quality of life

To measure the health-related quality of life (HRQoL) of the patients ≥12 years of age, the Dutch version of the EuroQol-5D (EQ-5D) was used. The EQ-5D index is obtained by applying predetermined weights to the five domains. This index gives a societal-based global quantification of the patient's health status on a scale that is anchored at 1 (which is perfect health). Patients were also asked to rate their overall HRQoL on a visual analogue scale (EQ-

5D VAS) consisting of a vertical line ranging from 0 (worst imaginable health status) to 100 (best imaginable). For children between 8 and 11 years of age the three level EQ-5D-youth was used. For children ≤7 years of age, parents were requested to fill in the EQ-5D-youth by proxy.

The first three months after the start of inclusion patients received the HRQoL questionnaire every four weeks, for a period of three months, after which the patient was asked to complete the questionnaire every three months. In case of disease recurrence or another (serious) adverse event, the EQ-5D was sent every four weeks for three months again to fully document the effect on quality of life during these periods.

Productivity losses

To measure health-care related productivity losses, the Medical Technology Assessment Productivity Costs Questionnaire (iPCQ) questionnaire was used. All patients ≥18 years of age received the iPCQ and all parents of patients <18 years of age received an adapted version of the iPCQ, to measure productively losses in families of patients with aHUS. Patients received the iPCQ questionnaire at start of inclusion and then every three months until the end of the study.

Supplementary Results S1 – Details of relapsing patients

Relapsing patients

Patient 5: this pediatric patient was been followed for a period of 24 months since eculizumab discontinuation. One relapse occurred 19 weeks after discontinuation, immediately following a viral triggering event. Due to frequent (viral) infections and age <6 years, eculizumab was continued at an tapered, 6-weekly interval in this patient (CH50 <10%).

Patient 11: this patient has been followed for a period of 53 months since first eculizumab discontinuation. In this patient, kidney function was still improving (≥10% difference in serum creatine (sCr) values) at time of eculizumab discontinuation, 43 days since start of therapy. Therefore, it cannot be excluded that kidney function could have improved to (some) further extent if eculizumab had been continued for the recommended 3 months. In addition, subtle signs of TMA (thrombocytes $142x10^9$/L, LDH 347 U/L, haptoglobin <0.3g/L, no AKI) were variably present during the first weeks after eculizumab discontinuation, while CH50 was ranging from <10% to >30%. A kidney biopsy revealed a proliferative C3 glomerulopathy (C3G), C3G was treated with prednisone, Mycofenolaat Mofetil (MMF), and one administration of eculizumab. Two aHUS relapses occurred 20 and 14 weeks after initial and second eculizumab discontinuation respectively. After the second relapse, eculizumab was continued on a tapered interval (CH50 <10%).

Patient 12: this patient has been followed for a period of 59 months since first eculizumab discontinuation. Thus far, 4 relapses of aHUS occurred always associated with 'febrile' episodes, likely reflecting viral infections. Meanwhile, these relapses have been treated successfully, the last relapse was treated for 8 weeks only. Patient is now 28 weeks after withdrawal of eculizumab, and will receive 'on demand' therapy from now on.

Patient 19: this patient has been followed for a period of 54 months since first eculizumab discontinuation. At time of eculizumab discontinuation (day 132), kidney function was not stable (≥10% difference in serum creatine (sCr) values) despite eculizumab treatment for >3 months. This patient developed two relapses, a first relapse 14 and a second relapse 12 weeks after eculizumab withdrawal. Currently, this patient is treated with eculizumab on a 6-weekly interval (CH50 >30%).

Self-limiting event without the need for eculizumab

Patient 17: In one patient TMA and AKI (increase of sCr from 223 to 410 µmol/L, thrombocytes $104x10^9$/L, LDH 423 U/L) occurred during flu-like symptoms and subsequent dehydration, however was self-limiting during close monitoring.

Supplementary Figure S1 – Treatment algorithm of the Dutch guideline in patients with (suspected) aHUS in native kidneys

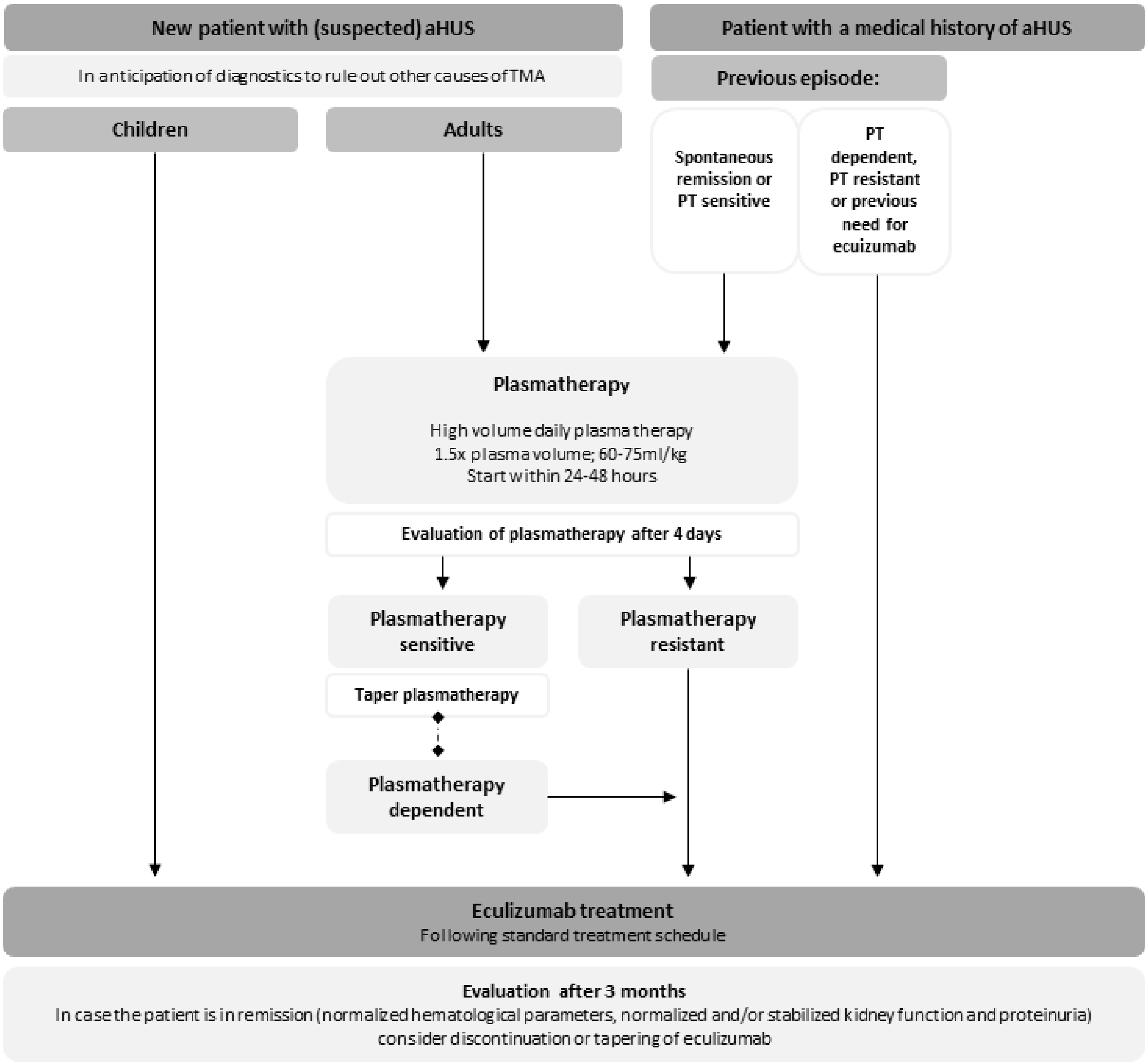

Supplementary Figure S2 – Individual utility scores over time

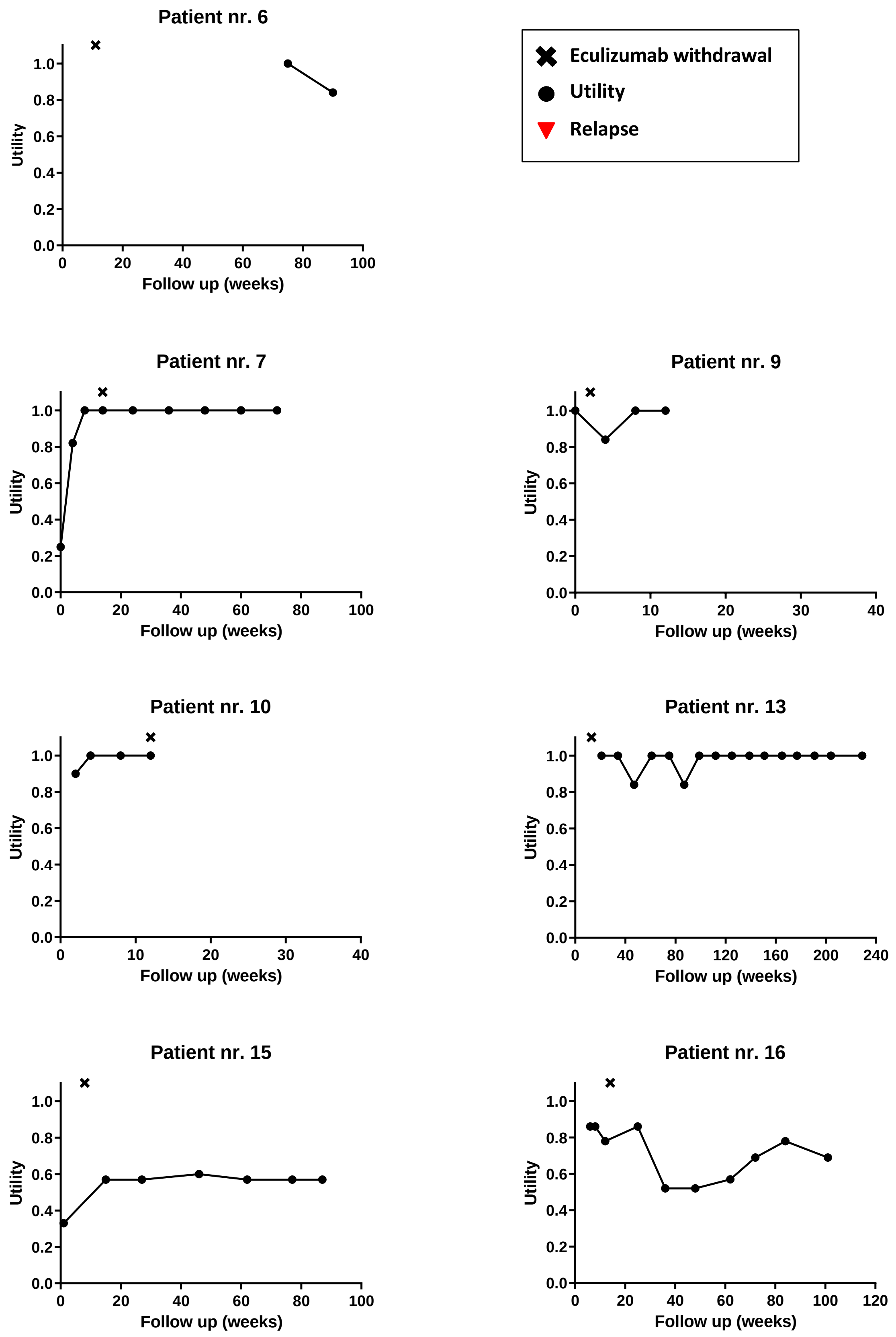

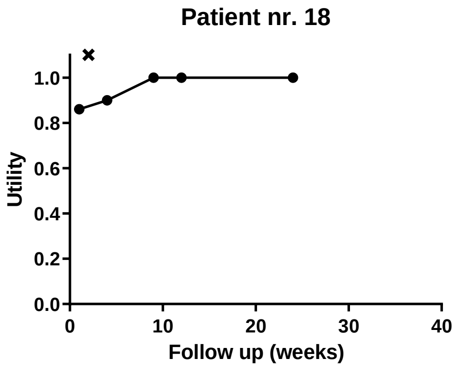

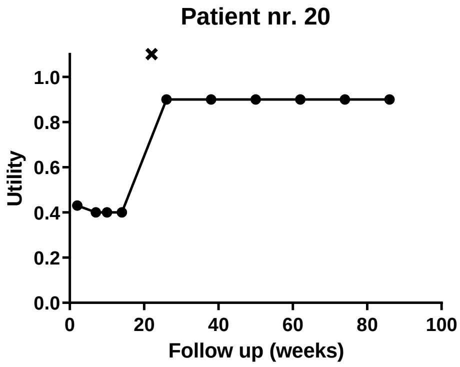

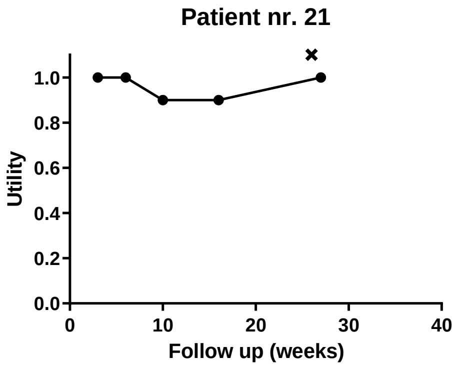

Relapsing patients

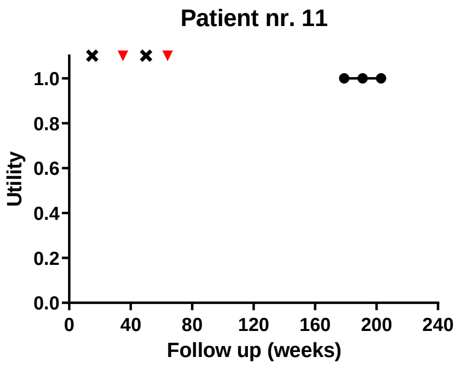

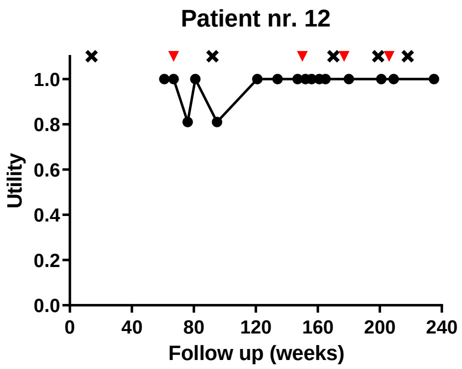

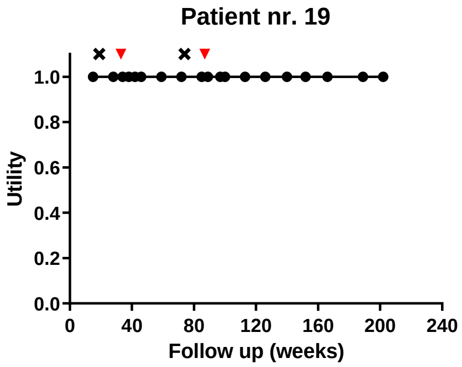

Supplementary Table S1 – genomic analysis of all patient

Patient nr.	Gene	cDNA level	Prot level	Heterozygous /Homozygous	MAF	Classification (class)	MCP*ggaac* haplotype homozygosity[4]	CFH-H3 haplotype homozygosity[5]	CFH auto antibodies	Missing
1	MCP<br>CFHR3-CFHR4 hybrid[1]	c.811_816del<br>-	p.Asp271_Ser272del<br>-	Heterozygous<br>Heterozygous	0.0004%<br>-	Pathogenic (V)[11]<br>-	Negative	Negative	Negative	-
2	-	-	-	-	-	-	Unknown	Negative	Positive	-
3	-	-	-	-	-	-	Unknown	Negative	Negative	-
4	CFHR3-1 deletion	-	-	Homozygous	-	-	Negative	Negative	Positive	CFHR1-5, THBD, DGKE
5	C3<br>CFI<br>DGKE	c.481C>T<br>c.392T>G<br>c.465-3C>T	p.Arg161Trp<br>p.Leu131Arg<br>-	Heterozygous<br>Heterozygous<br>Heterozygous	0.0004%<br>0.00071%<br>0.065%	Pathogenic (V)[12, 13]<br>Uncertain significance (III)[14]<br>Uncertain significance (III)	Positive	Negative	Negative	-
6	CFH-CFHR1 hybrid[2]	-	-	Heterozygous	-	Likely pathogenic (IV)	Negative	Negative	Negative	-
7	MCP	c.811_816del	p.Asp271_Ser272del	Heterozygous	0.0004%	Pathogenic (V)[11]	Negative	Negative	Negative	-
8	CFH	c.3644G>A	p.Arg1215Gln	Heterozygous	-	Likely pathogenic (IV)[15]	Negative	Positive	Negative	-
9	CFH-CFHR1 hybrid[2]	-	-	Heterozygous	-	Likely pathogenic (IV)	Negative	Negative	Unknown	-
10	C3	c.3760C>T	p.Arg1254cys	Heterozygous	0.0008%	Uncertain significance (III)	Negative	Negative	Unknown	-
11	C3	c.481C>T	p.Arg161Trp	Heterozygous	0.0004%	Pathogenic (V)[12,13]	All but c.−652A>G	Negative	Unknown	-
12	C3	c.481C>T	p.Arg161Trp	Heterozygous	0.0004%	Pathogenic (V)[12,13]	Unknown	Negative	Negative	CFHR1-5, THBD, DGKE
13	CFH	c.2850C>T	p.Gln950His	Heterozygous	0.39%	Likely benign (II)[16]	Unknown	Negative	Unknown	CFHR1-5, THBD, DGKE
14	CFH<br>MCP	c.1198C>A<br>c.811_816del	p.Gln400Lys<br>p.Asp271_Ser272del	Heterozygous<br>Homozygous	0.012%<br>0.0004%	Uncertain significance (III)<br>Pathogenic (V)[17]	Negative	Negative	Negative	-
15	-	-	-	-	-	-	Negative	Negative	Negative	-
16	C3	c.481C>T	p.Arg161Trp	Heterozygous	0.0004%	Pathogenic (V)[12,13]	Positive	Negative	Negative	-
17	CFI	c.1623G>T	Trp541Cys	Heterozygous	-	Uncertain significance (III)	Negative	Negative	Negative	CFHR1-5, THBD, DGKE
18	C3	c.481C>T	p.Arg161Trp	Heterozygous	0.0004%	Pathogenic (V)[12,13]	Positive	Negative	Negative	-
19	CFH rearrangement[3]	-	-	Heterozygous	-	Likely pathogenic (IV)	Negative	Negative	Negative	-
20	CFH<br>CFH<br>CFHR5	c.2850G>T<br>c.3616C>T<br>c.432A>T	Gln950His<br>p.Arg1206Cys<br>p.Lys144Asn	Heterozygous<br>Heterozygous<br>Heterozygous	0.39%<br>-<br>0.11%	Likely benign (II)[16]<br>Likely pathogenic (IV)[18]<br>Uncertain significance (III)	Negative	Negative	Unknown	-
21	-	-	-	-	-	-	Negative	Negative	Unknown	-

1 Genomic analysis using MLPA identified a heterozygous deletion of CFHR3 (LRG_175t1) exon 6, complete CFHR1 (LRG_149t1) and CFHR4 (LRG_1224t1) exons 1-8, indicating a genomic rearrangement resulting in a CFHR3/CFHR4 hybrid gene (NC_000001.10: g.(196758495_196762608)_(196883557_196886879)del).

2 Genomic analysis using MLPA identified a heterozygous deletion of CFH (LRG_47t1) exon 22, complete CFHR3 (LRG_175t1) and CFHR1 (LRG_149t1) exons 1-5, indicating a genomic rearrangement resulting in a CFH/CFHR1 hybrid gene (NC_000001.10: g.(196715167_196716320)_(196799888_196801005)del).

3 Genomic analysis using MLPA identified a heterozygous duplication with the probes targeting CFH (LRG_47t1) exons 21-22 and CFHR3 (LRG_175t1) exons 1-3 (NC_000001.10: g.(196711072-196715167)_(196749165-196758495)dup), and a heterozygous deletion with the probed targeting CFHR1 (LRG_149t1; NC_000001.10: g.(196762607_196796531)_(196801305_196913799)del). This probably indicates a heterozygous conversion of CFHR1 with a homologous region of CFH and CFHR3, but since we have no genetic data from the parents, this is only a hypothesis.

4 The risk haplotype MCP*ggaac* is formed by: c.−652A>G (rs2796267), c.−366A>G (rs2796268), c.IVS9−78G>A (rs1962149), c.IVS12+638G>A (rs859705), and c.4070T>C (rs7144)

5 The risk haplotype CFH-H3 is formed by: c.-331C>T (rs3753394), c.2016A>G (rs3753396), and c.2808G>T (rs1065489)

6 Genomic analysis using MLPA identified a heterozygous deletion of CFHR3 (LRG_175t1) exon 6, complete CFHR1 (LRG_149t1) and CFHR4 (LRG_1224t1) exons 1-8, indicating a genomic rearrangement resulting in a CFHR3/CFHR4 hybrid gene (NC_000001.10: g.(196758495_196762608)_(196883557_196886879)del).

7 Genomic analysis using MLPA identified a heterozygous deletion of CFH (LRG_47t1) exon 22, complete CFHR3 (LRG_175t1) and CFHR1 (LRG_149t1) exons 1-5, indicating a genomic rearrangement resulting in a CFH/CFHR1 hybrid gene (NC_000001.10: g.(196715167_196716320)_(196799888_196801005)del).

8 Genomic analysis using MLPA identified a heterozygous duplication with the probes targeting CFH (LRG_47t1) exons 21-22 and CFHR3 (LRG_175t1) exons 1-3 (NC_000001.10: g.(196711072-196715167)_(196749165-196758495)dup), and a heterozygous deletion with the probed targeting CFHR1 (LRG_149t1; NC_000001.10: g.(196762607_196796531)_(196801305_196913799)del). This probably indicates a heterozygous conversion of CFHR1 with a homologous region of CFH and CFHR3, but since we have no genetic data from the parents, this is only a hypothesis.

9 The risk haplotype MCP*ggaac* is formed by: c.−652A>G (rs2796267), c.−366A>G (rs2796268), c.IVS9−78G>A (rs1962149), c.IVS12+638G>A (rs859705), and c.4070T>C (rs7144)

10 The risk haplotype CFH-H3 is formed by: c.-331C>T (rs3753394), c.2016A>G (rs3753396), and c.2808G>T (rs1065489)

Abbreviations: DNA; Deoxyribonucleic Acid, MAF; Minor Allele Frequency

References:

11 Richards A, Kemp EJ, Liszewski MK, et al. Mutations in human complement regulator, membrane cofactor protein (CD46), predispose to development of familial hemolytic uremic syndrome. Proc Natl Acad Sci U S A. 2003 Oct 28;100(22):12966-71.

12 Roumenina LT, Frimat M, Miller EC, et al. A prevalent C3 mutation in aHUS patients causes a direct C3 convertase gain of function. Blood. 2012 May 3;119(18):4182-91.

13 Volokhina E, Westra D, Xue X, Gros P, van de Kar N, van den Heuvel L. Novel C3 mutation p.Lys65Gln in aHUS affects complement factor H binding. Pediatr Nephrol. 2012 Sep;27(9):1519-24.

14 de Jong S, Volokhina EB, de Breuk A, et al. Effect of rare coding variants in the CFI gene on Factor I expression levels. Hum Mol Genet. 2020 Aug 11;29(14):2313-2324.

15 Loeven MA, Rops AL, Lehtinen MJ, et al. Mutations in Complement Factor H Impair Alternative Pathway Regulation on Mouse Glomerular Endothelial Cells in Vitro. J Biol Chem. 2016 Mar 4;291(10):4974-81.

16 Martín Merinero H, Zhang Y, Arjona E, et al. Functional characterization of 105 factor H variants associated with aHUS: lessons for variant classification. Blood. 2021 Dec 2;138(22):2185-2201.

17 Richards A, Kemp EJ, Liszewski MK, et al. Mutations in human complement regulator, membrane cofactor protein (CD46), predispose to development of familial hemolytic uremic syndrome. Proc Natl Acad Sci U S A. 2003 Oct 28;100(22):12966-71.

18 Lehtinen MJ, Rops AL, Isenman DE, van der Vlag J, Jokiranta TS. Mutations of factor H impair regulation of surface-bound C3b by three mechanisms in atypical hemolytic uremic syndrome. J Biol Chem. 2009 Jun 5;284(23):15650-8.

Supplementary Table S2 – aHUS diagnosis classification of all patients

Patient nr.	Age at start study / Age of onset (years)	Sex	aHUS diagnosis classification
1	3.1	F	Definite
2	5.9	M	Definite
3	0.3	M	Possible, A
4	5.7	M	Definite
5	3.7	F	Definite
6	10.4	M	Definite
7	6.6	F	Definite
8	24.9	F	Definite
9	60.8 / 46.6	F	Definite
10	64.1	F	Definite
11	37.8	M	Definite
12	32.8	M	Definite
13	48.7	M	Possible, A
14	31.7 / 17.2	F	Definite
15	78.5	M	Possible, A
16	50.4	M	Definite
17	55.2	F	Definite
18	25.5 / 22.1	F	Definite
19	26.3	M	Definite
20	45.3	F	Definite
21	37.1	F	Possible, A

Supplementary Table S3 – Non-eculizumab treatment in patients with aHUS

	**Adults (n=14)**	**Children (n=7)**
**Acute phase, treatment**		
Dialysis (n;%)	8 (57.1%)	3 (42.9%)
Duration of temporary dialysis (days)[1]	28 (1-42)	11.5 (11-12)
Plasmatherapy (n;%)	14 (100%)	1 (14.3%)
Duration of plasmatherapy (days)	4 (1-50)	1 (-)
Interval from symptoms to start eculizumab (days)	14 (5-67)	5 (3-14)
Interval from diagnosis TMA to start eculizumab (days)	6 (2-63)	2 (1-11)

[1] In patients (6 adults, 2 children) who (partially) recovered kidney function

Abbreviation: TMA; thrombotic microangiopathy
Unless stated differently, median values and ranges are shown.

Supplementary Table S4 – Outcomes in aHUS patients after initial eculizumab treatment, discontinuation, and last follow-up

	**Native kidneys incident** (n;%)	
	**Adults (n=14)**	**Children (n=7)**
**Eculizumab response**		
Hematological remission[1]	14 (100%)	7 (100%)
Full kidney function recovery[2]	4 (28.6%)	6 (85.7%)
Duration to full kidney function recovery (days)	45 (29-67)	24 (6-32)
Partial kidney function recovery	8 (57.1%)	0 (0%)
Duration to partial kidney function recovery (days)	87 (15-1286)	-
No kidney function recovery	2 (14.3%)	1 (14.3%)
**Follow-up at eculizumab discontinuation[3]**		
Duration of eculizumab treatment (weeks)	13.7 (2.1-43.9)	13.0 (9.0-30.9)
Serum creatinine (µmol/L)[4]	142 (52-223)	37 (25-50)
eGFR (ml/min/1.73m^2)[4]	49 (24-90)	90 (90)
UPCR (g/10mmol)[4]	0.12 (0.05-1.55)[7]	0.18 (0.05-0.58)
Hypertension[5]	11 (78.6%)	1 (14.3%)
**Last follow-up[6]**		
Duration of follow-up after ecu discontinuation (weeks)	89.7 (0-222.7)	80.7 (7.3-236.9)
Serum creatinine (µmol/L)[4]	116 (60-268)	43.5 (34-60)
eGFR[4]	55 (19-90)	90 (90)
UPCR (g/10mmol)[4]	0.14 (0.04-0.53)[8]	0.16 (0.10-0.29)
Hypertension[5]	12 (85.7%)	3 (42.9%)

1 Improvement of TMA parameters: platelet count >150 x 10^9/L, lactate dehydrogenase (LDH) below the upper limit of normal (<250 U/l) and normal/detectable haptoglobin (>0.3 mg/L).

2 Including recovery to baseline, n=1. In relapsing patients, recovery to first relapse was included.

3 The following values were included (for serum creatinine, eGFR and UPCR): If 1] stable parameters (<10% change ≤30 days before or after discontinuation), mean value was included , or 2] unstable parameters (>10% change ≤30 days before or after discontinuation), lowest eGFR and highest sCR and UPCR values were included.

4 N=4 patients were excluded, three on chronic KRT and one adult patient who was on dialysis at time of eculizumab discontinuation but could stop dialysis soon after. At last follow-up eGFR in this patient was 10 ml/min/1.73m^2. Of note, this patient was at risk of relapse and thus included in the remaining analysis.

5 Defined as need for antihypertensive medication

6 The following values were included (for serum creatinine, eGFR and UPCR): If stable parameters 1] <10% change ≤30 days before last follow-up), mean value was included , or 2] unstable parameters (>10% change ≤30 days before or after discontinuation), lowest eGFR and highest sCR and UPCR values were included.

7 UPCR missing in 2 adult patients at time of eculizumab discontinuation

8 UPCR missing in 3 adult patients at time of last follow-up

Abbreviations: eGFR; estimated glomerular filtration rate, UPCR; urinary protein to creatinine ratio, CKD; chronic kidney disease, TMA; thrombotic microangiopathy, ESKD; end-stage kidney disease, KRT; kidney replacement therapy
Unless stated differently, median values and (min-max) ranges are shown.

Supplementary Table S5 – Outcomes at eculizumab discontinuation and last follow-up in relapsing patients

Patient nr.		**5**	**11**	**12**	**19**
**Eculizumab discontinuation**[1]					
**Serum creatinine**	*(µmol/l)*	34	179	90	142
**eGFR**	*(ml/min/1.73m^2)*	90	40	90	58
**UPCR**	*(g/10mmol)*	0.23	0.53	0.05	1.55
**Relapse(s)**					
**Max serum creatinine**	*(µmol/l)*	116	228 | 178	166 | 113<br>130 | 351	281 | 243
**Min eGFR**	*(ml/min/1.73m^2)*	36	36 | 41	46 | 72<br>60 | 18	25 | 30
**Max UPCR**	*(g/10mmol)*	23.4	5.59 | 0.94	0.40 | 0.52<br>1.81 | 0.37	- | 0.44
**Last follow-up**					
**Serum creatinine**	*(µmol/l)*	49	162	93	116
**eGFR**	*(ml/min/1.73m^2)*	90	45	90	72
**UPCR**	*(g/10mmol)*	0.29	0.04	0.12	0.27
**Eculizumab treatment**		Tapered,<br>6-weekly	Tapered,<br>4-weekly	Discontinued	Tapered,<br>6-weekly

[1] In case of multiple relapses, at first eculizumab discontinuation

Supplementary Table S6 – Characteristics and outcomes of relapsing versus non-relapsing patients (without ESKD)

	**No relapse (n=14)**	**Relapse(s) (n=4)**
**Sex**		
Female	8 (57.1%)	1 (25%)
Male	6 (42.9%)	3 (75%)
**Median age** (years)	28.6 (3.1-78.5)	29.5 (3.7-37.8)
Children	5 (35.7%)	1 (25%)
Adults	9 (64.3%)	3 (75%)
**Patients with a complement genetic variant**[1]	10 (71.4%)	4 (100%)
CFH[2]	6 (42.8%)[11]	1 (25%)
MCP	3 (21.4%)	0 (0%)
CFI	1 (7.1%)	1 (25%)
C3	3 (21.4%)	3 (75%)
CFB	0 (0%)	0 (0%)
Patients with >1 complement genetic variants[1]	2 (14.3%)	1 (25%)
**Anti-factor H antibodies**	2 (14.3%)	0 (0%)
**Patients with a complement genetic variant[1] and:**		
MCP*ggaac* haplotype homozygosity	2 (14.3%)	1 (25%)
CFH-H3 haplotype homozygosity	1 (7.1%)	0 (0%)
**Medical history**		
Previous aHUS episode[2]	3 (21.4%)	0 (0%)
Median age at aHUS onset (years)	23.5 (3.1-78.5)	29.5 (3.7-37.8)
aHUS Family history[3]	3 (21.4%)	2 (50%)
CKD[4] / Hypertension	3 (21.4%)	0 (0%)
**Eculizumab response**		
Hematological remission[5]	14 (100%)	4 (100%)
Full kidney function recovery[6]	8 (57.1%)	2 (50%)
Duration to full kidney function recovery (days)	28 (19-67)	30 (6-54)
Partial kidney function recovery	6 (42.9%)	2 (50%)
Duration to partial kidney function recovery (days)	70 (15-1286)	125 (90-159)
**Follow-up at eculizumab discontinuation**[7]		
Duration of eculizumab treatment (weeks)	13.2 (2.1-43.9)	16.3 (6.1-30.9)
Serum creatinine (µmol/L)[8]	64 (25-223)	116 (34-179)
eGFR (ml/min/1.73m^2)[8]	90 (24-90)	74 (40-90)
UPCR (g/10mmol)[8]	0.13 (0.05-0.58)[12]	0.38 (0.05-1.55)
Hypertension[9]	7 (50%)	3 (75%)
**Last follow-up**[10]		
Duration of follow-up after ecu discontinuation (weeks)	75.4 (0-236.9)	199.5 (65.0-222.7)
Serum creatinine (µmol/L)[8]	78 (34-268)	105 (49-162)
eGFR (ml/min/1.73m^2)[8]	90 (19-90)	81 (45-90)
UPCR (g/10mmol)[8]	0.15 (0.08-0.53)[13]	0.20 (0.04-0.29)
Hypertension[9]	9 (64.3%)	3 (75%)

1 Including complement gene class III rare variants of unknown significance (VUS), class IV variants (likely pathogenic), and class V variants (pathogenic)
2 Only aHUS episodes that were not treated with eculizumab
3 Family history was defined as at least one family member with a (officially diagnosed) medical history of aHUS. Unaffected family members with only a (likely) pathogenic variation (carriers) were not included
4 CKD defined as eGFR <60 ml/min/1.73m^2. Of all patients with a previous episode of TMA/aHUS, CKD was diagnosed in 2/3 (67%) prior to start of the CUREiHUS study. The remaining patient had CKD due to hydronephrosis and uncontrolled hypertension. Patient(s) with pre-existing ESKD were excluded.
5 Improvement of TMA parameters
6 Including recovery to baseline, n=1

[7] The following values were included (for serum creatinine, eGFR and UPCR): If 1] stable parameters (<10% change ≤30 days before or after discontinuation), mean value was included , or 2] unstable parameters (>10% change ≤30 days before or after discontinuation), lowest value was included.

[8] All patients were included, except one adult patient who was on dialysis at time of eculizumab discontinuation but could stop dialysis soon after. At last follow-up eGFR in this patient was 10 ml/min/1.73m^2. Of note, this patient was at risk of relapse and thus included in the remaining analysis.

[9] Defined as need for antihypertensive medication

[10] The following values were included (for serum creatinine, eGFR and UPCR): If stable parameters 1] <10% change ≤30 days before last follow-up), mean value was included , or 2] unstable parameters (>10% change ≤30 days before or after discontinuation), lowest value was included.

[11] Total of 5 patients with a CFH genetic variant. One patient had two CFH genetic variants.

[12] UPCR missing in 2 adult patients at time of eculizumab discontinuation

[13] UPCR missing in 3 adult patients at time of last follow-up

Abbreviations: eGFR; estimated glomerular filtration rate, UPCR; urinary protein to creatinine ratio, CKD; chronic kidney disease, TMA; thrombotic microangiopathy, ESKD; end-stage kidney disease, KRT; kidney replacement therapy

Unless stated differently, median values and (min-max) ranges are shown.

STROBE Statement—checklist of items that should be included in reports of observational studies

	Item No.	Recommendation	Page No.	Relevant text from manuscript
**Title and abstract**	1	(*a*) Indicate the study's design with a commonly used term in the title or the abstract	2	'prospective, observational study'
		(*b*) Provide in the abstract an informative and balanced summary of what was done and what was found	2	it is safe and (cost-) effective to discontinue eculizumab after three months of therapy in patients with aHUS in native kidneys.
**Introduction**				
Background/rationale	2	Explain the scientific background and rationale for the investigation being reported	3/4	In 2012, the complement C5-inhibitor eculizumab was approved for patients with atypical hemolytic uremic syndrome (aHUS)…. Furthermore, follow-up duration was relatively short in these studies
Objectives	3	State specific objectives, including any prespecified hypotheses	4	Therefore, we conducted a four-year prospective national multicentre study to assess the safety and cost-consequence of a restrictive eculizumab treatment strategy in an unselected cohort of patients with aHUS in native kidneys.
**Methods**				
Study design	4	Present key elements of study design early in the paper	4/5/7	prospective observational study/ genomic analysis was performed/ all suspected aHUS patients were categorized/ prospectively according to a restrictive eculizumab/ An

				extensive cost consequence analysis
Setting	5	Describe the setting, locations, and relevant dates, including periods of recruitment, exposure, follow-up, and data collection	4/5/6	conducted from 1-1-2016 to 1-10-2020 in all university medical centers in the Netherlands/ treated prospectively/ Regular follow-up at the outpatient/statistical analysis
Participants	6	(*a*) *Cohort study*—Give the eligibility criteria, and the sources and methods of selection of participants. Describe methods of follow-up<br>*Case-control study*—Give the eligibility criteria, and the sources and methods of case ascertainment and control selection. Give the rationale for the choice of cases and controls<br>*Cross-sectional study*—Give the eligibility criteria, and the sources and methods of selection of participants	4/5/6	Patients with… deficiency)/ In retrospect, all… excluded from analysis/ Follow-up
		(*b*) *Cohort study*—For matched studies, give matching criteria and number of exposed and unexposed<br>*Case-control study*—For matched studies, give matching criteria and the number of controls per case		
Variables	7	Clearly define all outcomes, exposures, predictors, potential confounders, and effect modifiers. Give diagnostic criteria, if applicable	7/8	Relapse/ Cost consequence analysis (among others, costs for eculizumab administrations, dialysis)/ health-related quality and EQ5D/ Glomerular filtration rate (eGFR) was estimated
Data sources/ measurement	8*	For each variable of interest, give sources of data and details of methods of assessment (measurement). Describe comparability of assessment methods if there is more than one group	*7/8*	Especially see Relapse and cost-consequence analysis and supplementary methods
Bias	9	Describe any efforts to address potential sources of bias	8/18	Due to a small sample size, EQ-5D utilities of patients with and without disease recurrence were presented for individual patients/ Our study had also some limitations… relapse
Study size	10	Explain how the study size was arrived at	Figure 1	See figure 1

Continued on next page

Quantitative variables	11	Explain how quantitative variables were handled in the analyses. If applicable, describe which groupings were chosen and why	20/5	Laboratory values were presented as quantitative data. For continuous variables, values were expressed using median and (min-max) range was used/ In retrospect, all suspected aHUS patients were categorized as *definite* aHUS, *possible* aHUS or *secondary* TMA after extensive evaluation of laboratory values and histology during the TMA episode and results of genetic analysis (Supplementary Method S1).
Statistical methods	12	(*a*) Describe all statistical methods, including those used to control for confounding	8	Statistical analysis
		(*b*) Describe any methods used to examine subgroups and interactions	8	'decriptively'
		(*c*) Explain how missing data were addressed	-	See subheading under tables if applicable
		(*d*) *Cohort study*—If applicable, explain how loss to follow-up was addressed<br>*Case-control study*—If applicable, explain how matching of cases and controls was addressed<br>*Cross-sectional study*—If applicable, describe analytical methods taking account of sampling strategy	-	Not applicable
		(*e*) Describe any sensitivity analyses	8	See Statistical analysis
**Results**				
Participants	13*	(a) Report numbers of individuals at each stage of study—eg numbers potentially eligible, examined for eligibility, confirmed eligible, included in the study, completing follow-up, and analysed	Figure 1	See figure 1 for exact numbers. Also given for each stage in (supplementary) tables.
		(b) Give reasons for non-participation at each stage	10	Overall, eculizumab was initiated for suspected aHUS in 46 patients… This analysis included, thus, 21 aHUS patients (14 adults and 7 children), who were treated with eculizumab.
		(c) Consider use of a flow diagram	Figure 1	Is a flow diagram
Descriptive data	14*	(a) Give characteristics of study participants (eg demographic, clinical, social) and information on exposures and potential confounders	Table 1	Characteristics of the patients are shown in Table 1. Genetic characteristics in supplementary file.
		(b) Indicate number of participants with missing data for each variable of interest	Tables	See subheading under every table

		(c) *Cohort study*—Summarise follow-up time (eg, average and total amount)	Figure 2	See for follow-up time of every participant
Outcome data	15*	*Cohort study*—Report numbers of outcome events or summary measures over time	10-11-12	See results (including subheadings): eg: In 18 (86%) patients, a defect in complement regulation/ Eleven patients (52%) needed kidney replacement therapy/ A first relapse occurred in 4/ During follow-up, 10 (S)AEs other than (related to the) aHUS relapses were registered
		Case-control study—Report numbers in each exposure category, or summary measures of exposure		
		Cross-sectional study—Report numbers of outcome events or summary measures		
Main results	16	(*a*) Give unadjusted estimates and, if applicable, confounder-adjusted estimates and their precision (eg, 95% confidence interval). Make clear which confounders were adjusted for and why they were included		Median (including ranges) were provided for all group measurements. For the cost-consequence analysis, mean values were included (as appropriate).
		(*b*) Report category boundaries when continuous variables were categorized	7 (among others)	Provided if applicable, eg: thrombocytopenia (platelet count <150 x 10^9/L),
		(*c*) If relevant, consider translating estimates of relative risk into absolute risk for a meaningful time period	NA	

Continued on next page

Other analyses	17	Report other analyses done—eg analyses of subgroups and interactions, and sensitivity analyses		Cost-consequence analysis.
**Discussion**				
Key results	18	Summarise key results with reference to study objectives	15/16	This is the first prospective, observational study demonstrating the safety of unbiased, controlled… who relapsed twice
Limitations	19	Discuss limitations of the study, taking into account sources of potential bias or imprecision. Discuss both direction and magnitude of any potential bias	18	Our study had also some limitations.
Interpretation	20	Give a cautious overall interpretation of results considering objectives, limitations, multiplicity of analyses, results from similar studies, and other relevant evidence	16/.17	Our data extends and strengthens the findings of other published aHUS cohorts > see following text/ see page 17 for comparison with one study with contrary results (The safety of eculizumab withdrawal was questioned in one study)
Generalisability	21	Discuss the generalisability (external validity) of the study results	18/19	demonstrates the safety and (cost-)effectiveness of eculizumab withdrawal after three months of therapy in well-defined, pediatric and adult patients with aHUS in native kidneys. Our results emphasize the feasibility.. Clinician-directed discontinuation (including adherence to protocol and discontinuation conditional upon stabilized kidney function), close monitoring, and patient collaboration appear to be a prerequisite for the safety of restrictive eculizumab management
**Other information**				
Funding	22	Give the source of funding and the role of the funders for the present study and, if applicable, for the original study on which the present article is based	32	This work was supported by grants from Zorgverzekeraars Nederland and ZonMw, ‘Goed Gebruik Geneesmiddelen’ (project number 836031008). They did not have any

			role in data collection, analysis or submission of this manuscript.

*Give information separately for cases and controls in case-control studies and, if applicable, for exposed and unexposed groups in cohort and cross-sectional studies.

Note: An Explanation and Elaboration article discusses each checklist item and gives methodological background and published examples of transparent reporting. The STROBE checklist is best used in conjunction with this article (freely available on the Web sites of PLoS Medicine at http://www.plosmedicine.org/, Annals of Internal Medicine at http://www.annals.org/, and Epidemiology at http://www.epidem.com/). Information on the STROBE Initiative is available at www.strobe-statement.org.
